# Effects of Task-Oriented Circuit Training on Dizziness, Vertigo Balance, Gait, and Quality of Life in Patients with Peripheral Vestibular Hypofunction: A Single-Blind, Randomized Controlled Trial

**DOI:** 10.3390/healthcare14060762

**Published:** 2026-03-18

**Authors:** Yasemin Apaydin, Çağla Özkul, Arzu Guclu-Gunduz, Umut Apaydin, Emre Orhan, Burak Kabiş, Ebru Şansal, Hakan Tutar, Bulent Gunduz

**Affiliations:** 1 Department of Neurological Physiotherapy and Rehabilitation, Guneysu Vocational School of Physical Therapy and Rehabilitation, Recep Tayyip Erdogan University, Rize 53600, Turkey; 2 Department of Physiotherapy and Rehabilitation, Faculty of Health Sciences, Gazi University, Ankara 06560, Turkey; caglaozkul@hotmail.com (Ç.Ö.); arzuguclu@hotmail.com (A.G.-G.); 3 Department of Physiotherapy and Rehabilitation, Faculty of Health Sciences, Karadeniz Technical University, Trabzon 61080, Turkey; umut.apaydin@ktu.edu.tr; 4 Department of Audiology, Faculty of Health Sciences, Gazi University, Ankara 06560, Turkey; emreorhan@gazi.edu.tr (E.O.); burakkabis@gmail.com (B.K.); bulentgunduz4@gazi.edu.tr (B.G.); 5 Department of Otolaryngology, Faculty of Medicine, Gazi University, Ankara 06560, Turkey; ebrusansal@gmail.com (E.Ş.); drhakantutar@gmail.com (H.T.)

**Keywords:** peripheral vestibular hypofunction, task-oriented circuit training, balance, gait, dizziness, quality of life, vestibular rehabilitation

## Abstract

**Background/Objectives:** Peripheral vestibular hypofunction (PVH) commonly causes dizziness, imbalance, gait disturbances, and reduced quality of life. Task-oriented circuit training (TOCT) is a rehabilitation approach in which patients perform structured, task-specific functional movements repetitively to improve real-life motor performance. TOCT integrates functional, multisensory, and repetitive exercises based on motor learning and neuroplasticity principles, potentially enhancing rehabilitation outcomes. This study aimed to investigate the effects of TOCT on dizziness, vertigo, balance, gait, disability, and quality of life in patients with PVH. **Methods:** In this single-blind, randomized controlled trial, 28 patients with PVH were randomly allocated to either a task-oriented circuit training (TOCT) group (n = 16) or a control group (n = 12). The control group performed a conventional home-based vestibular exercise program consisting of gaze stabilization and walking exercises. The TOCT group completed 25 task-specific stations, targeting gaze stabilization, balance, and gait, three times per week for four weeks. Outcomes were assessed at baseline and post-intervention using the Visual Analog Scale for dizziness and vertigo, the Sensory Organization Test for balance, spatiotemporal gait analysis, and the Dizziness Handicap Inventory (DHI) for disability and quality of life. Data were analyzed using two-way repeated-measures ANOVA, with the group × time interaction used to determine whether changes over time differed between the TOCT and control groups. **Results:** Significant time × group interactions favored TOCT for dizziness severity, vertigo severity, vestibular-related balance parameters, cadence during eyes-closed walking, and DHI total scores (*p* < 0.05). Within-group analyses demonstrated moderate-to-large improvements in all measured outcomes for the TOCT group, whereas the control group showed limited improvements in dizziness measures and minimal changes in balance, gait, and DHI scores. **Conclusions:** Task-oriented circuit training significantly improves dizziness, vertigo, balance, gait, disability, and overall quality of life in patients with PVH compared with conventional home-based vestibular exercises. Incorporating functional, multisensory, and task-specific activities within structured circuits may optimize vestibular rehabilitation outcomes.

## 1. Introduction

Peripheral vestibular hypofunction (PVH) is a heterogeneous disorder characterized by reduced or absent function of one or both peripheral vestibular organs and/or the vestibular nerves. This impairment disrupts the processing of head movement and spatial orientation signals, resulting in symptoms such as dizziness, vertigo, imbalance, oscillopsia, postural instability, gait disturbances, and an increased risk of falls. These symptoms may substantially limit daily activities and reduce quality of life. PVH may result from various etiologies, including vestibular neuritis, trauma, surgical transection, ototoxic medications, Ménière’s disease, vestibular nerve lesions, infectious or autoimmune diseases, vascular disorders, genetic abnormalities, or congenital causes. However, the underlying etiology remains unidentified in more than 50% of cases [1]. Epidemiological studies indicate that vestibular dysfunction affects approximately 35.4% of adults aged 40 years and older in the United States, with prevalence increasing markedly with age [2].

Management of vestibular hypofunction includes medical treatment, surgical interventions, and vestibular rehabilitation. Pharmacological treatment is generally used for symptom control, particularly for nausea and vertigo during the acute phase, or to address specific underlying conditions. Surgical procedures may be indicated in selected cases such as vestibular organ repair or removal of lesions including acoustic neuroma [3,4]. However, these approaches are not always sufficient to restore functional balance or mobility. Therefore, vestibular rehabilitation is the primary treatment approach for patients with PVH [1]. Its principles were first introduced by Cawthorne and Cooksey in the 1940s and have since been widely applied in clinical practice [1,3,4]. Vestibular rehabilitation has been shown to improve balance, gaze and postural stability, activities of daily living, walking performance, and quality of life, while reducing dizziness, disability, and fall risk [1]. Although vestibular rehabilitation is well established as an exercise-based intervention for vestibular disorders, emerging neurophysiological approaches grounded in motor learning and central nervous system neuroplasticity may further enhance rehabilitation outcomes [5,6,7].

Task-oriented circuit training (TOCT) is a rehabilitation approach based on movement science and motor learning principles, in which patients practice task-specific, functional movements in structured circuits. Rather than targeting isolated impairments, TOCT emphasizes improving functional task performance through high repetition, variable practice conditions, contextual relevance, and feedback. Training is typically conducted in real or task-similar environments, promoting the transfer of learned motor strategies to daily activities [5]. Previous studies have primarily applied TOCT in neurological populations, including individuals with multiple sclerosis, Parkinson’s disease, and stroke [6,7,8,9]. Evidence from randomized and clinical studies indicates that TOCT can improve motor and cognitive performance in patients with multiple sclerosis and enhance motor performance and balance when combined with aerobic training in patients with Parkinson’s disease [6,7]. In patients with subacute stroke, TOCT has also been shown to improve walking ability [8]. Additionally, a three-month home-based program incorporating high-intensity TOCT was reported to be feasible and safe for patients with moderate mobility limitations due to multiple sclerosis and was associated with improved walking performance [9]. Collectively, these findings suggest that TOCT is a promising rehabilitation strategy for improving functional outcomes in neurological populations and may also help address the core functional impairments observed in PVH.

The symptoms experienced by patients with PVH often lead to movement avoidance, sedentary behavior, and restrictions in daily activities such as walking, standing, and household tasks [10,11,12]. This activity avoidance may further impair vestibular compensation and functional recovery. Repeated practice of symptom-provoking functional activities is considered essential for vestibular recovery. While symptoms may improve rapidly in the acute phase following vestibular injury due to central compensation, chronic vestibular hypofunction is characterized by reduced vestibular afferent input and impaired dynamic reflex function. Consequently, vestibular rehabilitation should aim to enhance peripheral sensory input and promote synaptic plasticity through repetitive, task-specific stimulation of the vestibular system [13]. In this context, TOCT—by emphasizing high-repetition practice of functional tasks in real-life environments—appears well aligned with the therapeutic requirements of vestibular rehabilitation. However, to date, no study has investigated the effects of TOCT in patients with PVH.

Therefore, the primary aim of this study was to investigate the effects of task-oriented circuit training on dizziness and vertigo in patients with PVH. The secondary aim was to examine its effects on balance, gait, disability level and quality of life.

We hypothesized that TOCT would lead to greater improvements in severity and frequency of dizziness and vertigo, balance, and gait performance compared with conventional vestibular rehabilitation, and would additionally result in reduced disability and improved quality of life in patients with PVH.

## 2. Materials and Methods

### 2.1. Participants

An otorhinolaryngologist performed the clinical examination of the patients with PVH, and two expert audiologists performed audiological examinations. The participants were recruited between June 2020 and December 2021 at Gazi University’s Department of Physiotherapy and Rehabilitation, Neurorehabilitation Outpatient Clinic. Inclusion criteria were: (1) receiving a diagnosis of peripheral vestibular hypofunction by an otorhinolaryngologist; (2) age between 18 and 65 years; (3) not having an inability to prevent exercise in physical functions; (4) not having orthopedic, neurological, rheumatological, etc., disease, which may cause balance disorder; (5) having peripheral-type dizziness, central pathologies excluded by videonystagmography, and findings supporting peripheral vestibular hypofunction in at least two of the following tests: videonystagmography, video head impulse, vestibular evoked myogenic potential and caloric tests. Patients were excluded if they had (1) cognitive dysfunction that may affect the research results; (2) a history of cerebrovascular accident, fainting, or epilepsy; (3) benign paroxysmal positional vertigo; (4) vestibular rehabilitation program in the last one month; (5) acute phase of vestibular hypofunction; (6) taken vestibular suppressants or centrally acting drugs in the last three months. All intervention sessions and outcome assessments were conducted at the Neurological Physiotherapy Unit of the Department of Physiotherapy and Rehabilitation, Faculty of Health Sciences, Gazi University, Ankara, Türkiye.

Before beginning the recruitment, the study was approved by the Non-Interventional Clinical Research Ethics Committee of Gazi University (Decision No: 309). All the patients were told about the study and provided written informed consent. The study was conducted in accordance with the principles of the Declaration of Helsinki and was registered at ClinicalTrials.gov with the identifier NCT06019104. A priori sample size calculation was performed using G*Power software (version 3.1.9.2, Universität Kiel, Germany). The calculation was based on detecting a medium effect size (f = 0.25; corresponding to η^2^p = 0.06) for the group × time interaction in a two-way repeated-measures ANOVA design with two groups and two measurement time points. The analysis assumed a statistical power of 0.90 (1 − β = 0.90) and a two-tailed significance level of α = 0.05 according to Cohen’s recommendations. The calculation was primarily based on the expected change in vertigo and dizziness severity measured using the Visual Analog Scale (VAS), which was considered the primary clinical outcome of the study. Based on these parameters, the required minimum sample size was determined to be 24 participants (12 per group) [14].

### 2.2. Study Design

The study was designed as a single-blind, randomized controlled trial. A total of 91 patients with PVH were assessed for eligibility. Of these, 52 patients were excluded, including 32 who did not meet the inclusion criteria and 20 who declined to participate. Consequently, 39 participants were randomized.

Participants were allocated to the task-oriented circuit training group (TOCTG, n = 19) or the control group (CG, n = 20) using an adaptive randomization procedure based on the minimization method to ensure balance between groups with respect to age and gender [15].

During the follow-up period, 3 participants in the TOCTG and 8 participants in the CG were lost to follow-up due to discontinuation of the intervention or incomplete post-intervention assessments. Therefore, the final per-protocol analysis included 16 participants in the TOCTG and 12 participants in the CG [15].

**Figure 1:**
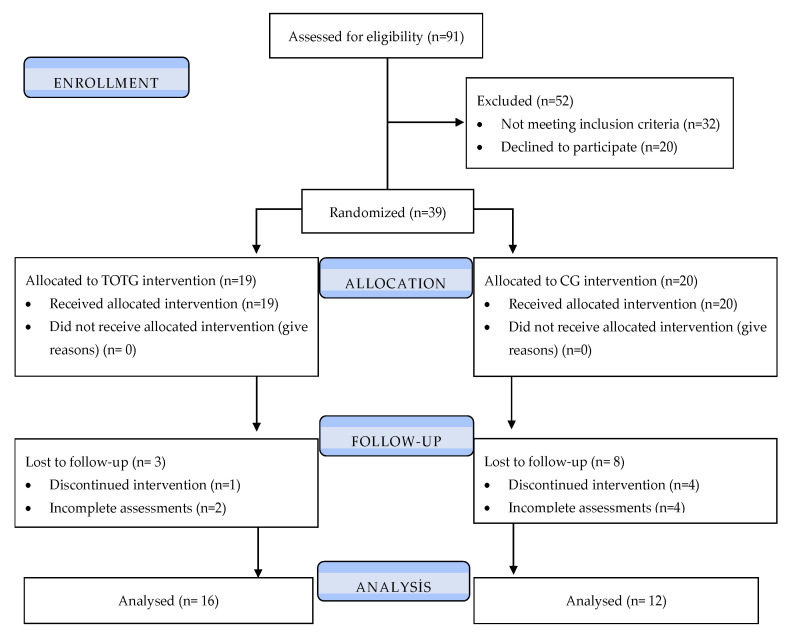
CONSORT flow diagram illustrating the patients participating in the study. CG: Control group; TOTG: Task-oriented training group.

Data collection and statistical analysis were performed by researchers who were not involved in the randomization process. All patients were evaluated by two researchers who were blinded to group allocation at baseline (T1), and four weeks later (T2). During the evaluation procedure, participants were instructed not to disclose their intervention to the researchers. The research design is shown in the Consolidated Standards of Reporting Trials (CONSORT) diagram (Figure 1).

### 2.3. Intervention Group

Participants in the TOCTG attended supervised training sessions three times per week for four weeks, resulting in a total of 12 sessions. The task-oriented circuit training comprised 25 stations, including nine gaze stabilization stations, seven balance stations, and nine gait training stations (Table 1).

Gaze stabilization exercises were initiated in a seated position on a Swiss ball and progressed to standing according to the participant’s tolerance. Balance and gait training stations were performed exclusively standing. Each task was performed for one minute with systematically alternated sensory inputs (somatosensory, vestibular, and visual), followed by a one-minute rest period. Accordingly, the total duration of each session was approximately 50 min. Training intensity and task difficulty were individualized and progressed based on perceived exertion, which was assessed after each session using the Borg Rating of Perceived Exertion (RPE) scale. The physiotherapist adjusted task difficulty to maintain an exertion level between 11 and 14 (mild to relatively hard), ensuring a standardized yet patient-specific training load [16,17].

The task-oriented circuit training was delivered using different clinical equipment, including balance pads, foam surfaces, BOSU balls, trampolines, balance discs, visual targets and screens, obstacle bars, cones, stairs, treadmills, rotating discs, and various objects for ground-level reaching and picking-up tasks. Task-related progression was implemented by systematically modifying predefined task parameters, including movement speed, range of head movements, base of support, surface stability, and visual conditions (from eyes open to eyes closed). To ensure intervention standardization, all sessions were conducted according to a predefined and structured protocol specifying task duration, rest intervals, progression criteria, and safety procedures. Therapist supervision was provided throughout all sessions. Therapist feedback was standardized and limited to safety monitoring and task-specific instructional cues. No physical facilitation or manual assistance was provided unless required to prevent loss of balance and ensure participant safety.

The frequency and duration of the intervention were selected in accordance with previous task-oriented circuit training studies and established vestibular rehabilitation protocols aimed at promoting vestibular adaptation and functional recovery.

### 2.4. Control Group

Participants in the CG performed a home-based basic exercise program three times per week for four weeks, resulting in a total of 12 sessions. The control program was designed to reflect commonly prescribed basic home-based vestibular exercises. It consisted of gaze stabilization exercises and walking training. The gaze stabilization program included head-fixed eye movements and eye-fixed head movements in the horizontal and vertical planes, as well as pursuit and saccadic eye movements. Depending on the participants’ clinical status, gaze stabilization exercises were initiated in a seated position and progressed to standing as tolerated. During the exercises, wall-mounted visual targets and foam surfaces were used. Exercise difficulty was adjusted by modifying movement speed, range of head movements, base of support, and surface stability. Exercise intensity was monitored using the Borg RPE scale during weekly telephone follow-ups, and the target exertion level was maintained between 11 and 14. Each gaze stabilization exercise was performed continuously for 2 min without rest, and the total duration of the gaze stabilization component was approximately 20 min.

Following the gaze stabilization exercises, participants completed a 30 min walking exercise on a flat surface at a self-selected comfortable pace. No additional head movements, dual-task activities, or balance-challenging tasks were included during walking. The total duration of each session was approximately 50 min. Adherence to the exercise program was monitored through a weekly telephone follow-up.

The main difference between the two interventions lies in the structure, complexity, and functional integration of the exercises. While the control group performed basic vestibular exercises in a home-based format, the TOCT group participated in a supervised, task-oriented circuit training program consisting of 25 functional stations targeting gaze stabilization, balance, and gait under varying sensory conditions. TOCT integrates multisensory stimulation, task-specific practice, and progressive functional challenges designed to simulate real-life motor activities, whereas the conventional program primarily focuses on isolated gaze stabilization and walking exercises.

### 2.5. Measurements

#### 2.5.1. Baseline Characteristics

All patients’ demographic information, including age, gender, body mass index, education level, and self-reported dominant side, as well as disease-related characteristics including disease duration and affected ear, were recorded at baseline.

#### 2.5.2. Primary Outcomes

Dizziness and vertigo severity and frequency were assessed using a numeric Visual Analog Scale. The same scale was used to evaluate both dizziness and vertigo. The severity of dizziness/vertigo during the previous seven days was scored as follows: 0–1 (no dizziness/vertigo), 2–3 (slight), 4–5 (moderate), 6–7 (severe), 8–9 (intense), and 10 (unbearable). The frequency of dizziness/vertigo during the previous seven days was graded as 0–1 (no dizziness/vertigo), 2–3 (1–5 times a week), 4–5 (1–3 times a day), 6–7 (4–10 times a day), 8–9 (>10 times a day), and 10 (always) [18].

#### 2.5.3. Secondary Outcomes

##### Balance Assessment

Balance was assessed using the Sensory Organization Test (SOT) with a computerized posturography system (Synapsis Posturographic System, software version 3.0, REV C). The SOT comprises six standardized sensory conditions designed to evaluate the relative contributions of somatosensory, visual, and vestibular inputs to postural control. The six SOT conditions were: (1) eyes open on a fixed support surface; (2) eyes closed on a fixed support surface; (3) eyes open on a fixed support surface with a sway-referenced visual surround; (4) eyes open on a sway-referenced support surface; (5) eyes closed on a sway-referenced support surface; (6) eyes open on a sway-referenced support surface with a sway-referenced visual surround. A firm surface and a foam surface were used to create fixed and sway-referenced support surface conditions, respectively. All SOT conditions were administered in the standard order according to the manufacturer’s protocol and under therapist supervision to ensure participant’s safety. Participants were instructed to stand as still as possible on the force platform for 20 s in each condition. Each condition was performed twice, and the mean of the two trials was used for statistical analysis. Postural sway was automatically calculated by the system, and equilibrium scores were obtained for each SOT condition. Scores ranged from 0 to 100, with higher scores indicating better postural stability and lower scores indicating increased postural sway. Somatosensory, visual, and vestibular ratios were calculated according to standard SOT algorithms in both the anteroposterior and mediolateral directions [19,20].

##### Gait Assessment

Gait was assessed using the BTS G-Walk^®^ spatiotemporal gait analysis system (G Sensor, BTS Bioengineering S.p.A., Milan, Italy). The device was secured at the L5–S1 level using a belt, and gait data were transmitted wirelessly to a computer via Bluetooth [21,22]. The system provided spatiotemporal gait parameters and three-dimensional kinematic analysis of pelvic motion during walking by capturing movement along the anteroposterior, mediolateral, and vertical axes. An eight-meter unobstructed corridor was used for gait assessment. The analysis was performed in a quiet environment with comfortable sneakers that the patients wore daily, and they were asked to wear the same shoes after the treatment. During the evaluation, walking tasks were performed under four conditions: eyes open, eyes closed, horizontal head movements, and vertical head movements. Each condition was repeated three times, and the mean of the three trials was used for analysis. Participants were instructed to walk as normally as possible at a self-selected pace. Spatiotemporal gait parameters, including gait speed and cadence, were recorded and used for analysis.

##### Disability and Quality of Life

The Dizziness Handicap Inventory (DHI) was used to assess dizziness-related disability and quality of life. The DHI evaluates the functional, physical, and emotional effects of dizziness over the previous month. The questionnaire consists of 25 items divided into three subscales: Physical (7 items), Emotional (9 items), and Functional (9 items). Each item is scored using three response options (“yes” = 4, “sometimes” = 2, and “no” = 0). The maximum possible scores are 28 for the Physical subscale and 36 for both the Emotional and Functional subscales, yielding a total maximum score of 100. Total DHI scores are interpreted as follows: 0–30 indicates mild disability, 31–60 indicates moderate disability, and 61–100 indicates severe disability [23]. Higher scores indicate greater dizziness-related disability and reduced quality of life [24].

### 2.6. Statistical Analysis

All statistical analyses were performed using SPSS software (version 26.0; IBM Corp., Chicago, IL, USA). Normality assumptions were evaluated using both statistical tests and graphical methods (histograms and Q–Q plots). Continuous variables are presented as mean ± standard deviation (SD), and categorical variables are presented as frequencies and percentages. Baseline demographic and clinical characteristics were compared between groups using the independent samples *t*-test for continuous variables and the chi-square test for categorical variables. Within-group comparisons between baseline (T1) and post-intervention (T2) were performed using the paired samples *t*-test. Effect sizes for within-group changes were calculated using Cohen’s d and interpreted as small (0.2), medium (0.5), or large (0.8) [25]. To examine between-group differences over time, a two-way repeated-measures analysis of variance (ANOVA) was conducted with time (T1, T2) as the within-subject factor and group (TOCTG, CG) as the between-subject factor. To verify the assumption relevant to the interaction term in the two-way repeated-measures ANOVA, the homogeneity of variance of the change scores (post–pre) between groups was assessed using Levene’s test. The results indicated that the assumption was not violated (*p* > 0.05). The interaction effect (time × group) was used to determine differential treatment effects between groups. When a significant interaction was observed, post hoc pairwise comparisons with appropriate adjustment for multiple comparisons were performed. Effect sizes for ANOVA results were reported using partial eta squared (η^2^p) and interpreted as small (0.01), medium (0.06), or large (0.14) [14]. Due to missing post-intervention outcome data for participants who withdrew from the study, analyses were conducted using a per-protocol approach including only participants who completed both baseline and post-intervention assessments. The level of statistical significance was set at *p* < 0.05.

## 3. Results

The baseline demographic and clinical characteristics of the participants were presented in Table 2. There were no statistically significant differences between the TOCTG and the CG in terms of age, body mass index (BMI), or disease duration (*p* > 0.05 for all). The mean age was 44.87 ± 9.32 years in the TOCTG and 45.66 ± 15.95 years in the CG, while BMI values were 28.01 ± 4.41 kg/m^2^ and 27.86 ± 5.63 kg/m^2^, respectively. Disease duration was comparable between groups (13.88 ± 13.04 months in TOCTG vs. 17.67 ± 18.91 months in CG). Regarding categorical variables, no significant differences were observed between groups in gender distribution, education level, dominant side, or affected side (*p* > 0.05). In the TOCTG, 56.3% of participants were female and 43.7% were male, while in the CG, 58.3% were female and 41.7% were male. Educational status, dominant side, and affected side distribution (right unilateral, left unilateral, or bilateral involvement) were also comparable between groups, indicating similar baseline clinical characteristics.

**Figure 2:**
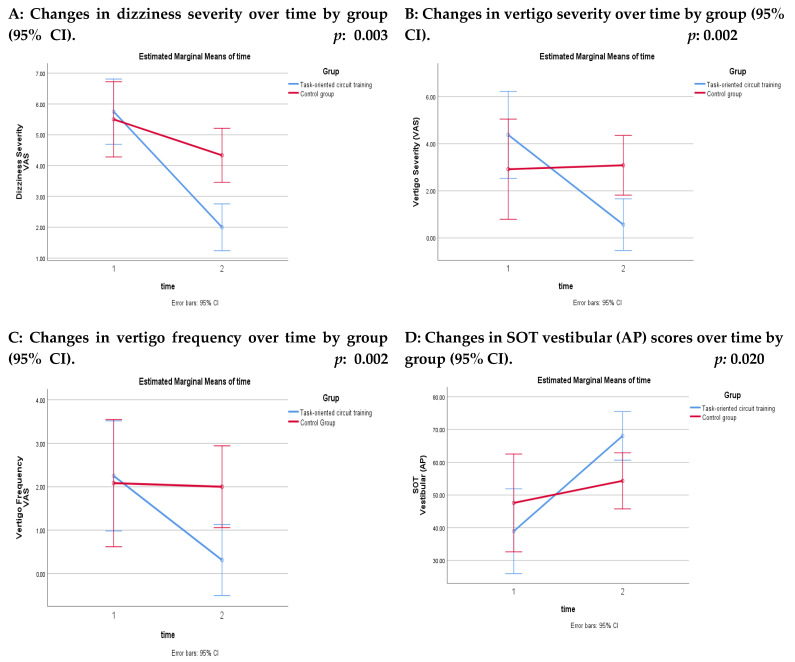
Comparisons of treatment and control groups (Note: Statistically significant time × group interactions are displayed graphically in Figure 2).

No significant between-group differences were observed at baseline in terms of dizziness severity, dizziness frequency, vertigo severity, or vertigo frequency (*p* > 0.05, these data were not presented in the tables). At four weeks, the time × group interaction effects revealed significant differences in dizziness severity, vertigo severity, and vertigo frequency in favor of the TOCTG (*p* < 0.05) (Table 3) (Figure 2). Within-group analyses demonstrated significant improvements with moderate to large effect sizes in dizziness severity, dizziness frequency, vertigo severity, and vertigo frequency in the TOCTG (*p* < 0.05). In the CG, significant within-group improvements with moderate to large effect sizes were observed in dizziness severity and dizziness frequency (*p* < 0.05) (Table 3).

No significant between-group differences were observed at baseline in balance scores measured by posturography (*p* > 0.05; these data were not presented in the tables). After four weeks, a significant time × group interaction was observed in favor of the TOCTG for the vestibular (AP) parameter (*p* < 0.05) (Table 4) (Figure 2). Within-group analyses demonstrated significant improvements with moderate to large effect sizes in somatosensory (AP), visual (AP), vestibular (AP), visual medial–lateral (ML), and vestibular (ML) parameters in the TOCTG (*p* < 0.05). In contrast, the CG showed no significant within-group changes, except for a significant improvement in the vestibular (ML) parameter (*p* < 0.05) (Table 4).

**Figure 3:**
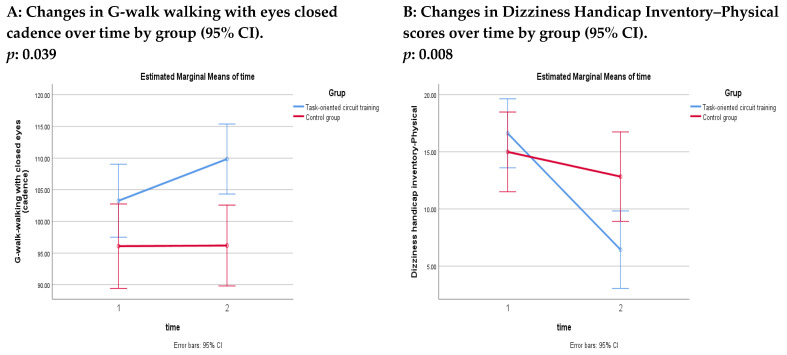

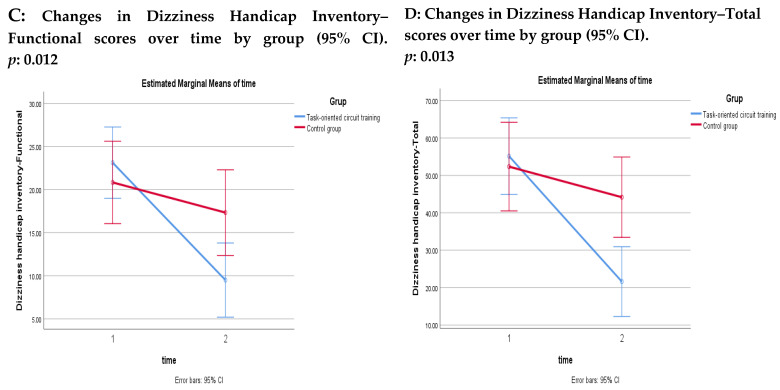
Comparisons of treatment and control groups (Note: Statistically significant time × group interactions are displayed graphically in Figure 3).

No significant between-group differences were observed at baseline in gait parameters (*p* > 0.05; these data were not presented in the tables). After four weeks, a significant time × group interaction was observed for cadence during walking with eyes closed, in favor of the TOCTG (*p* < 0.05) (Table 5) (Figure 3). Within-group analyses demonstrated significant improvements with moderate to large effect sizes in cadence during walking with eyes open and eyes closed, walking with horizontal head movements, and walking with vertical head movements in the TOCTG (*p* < 0.05). In contrast, the CG showed no significant within-group changes, except for significant improvements in cadence and gait speed during walking with vertical head movements (*p* < 0.05) (Table 5).

No significant between-group differences were observed at baseline in DHI total or subscale scores (*p* > 0.05; these data were not presented in the tables). At four weeks, significant time × group interaction effects were observed in favor of the TOCTG for all DHI outcomes, except for the DHI-Emotional subscale (*p* < 0.05) (Table 6) (Figure 3). Within-group analyses demonstrated significant improvements in the TOCTG for the DHI total score and all subscale scores (*p* < 0.05). In contrast, no significant within-group changes were observed in any DHI scores in the CG (*p* > 0.05) (Table 6).

## 4. Discussion

This randomized controlled trial examined the effects of TOCT on dizziness, vertigo, balance, gait, disability level, and quality of life in patients with PVH. The main findings demonstrated significant time × group interaction effects in favor of TOCT for dizziness and vertigo outcomes, vestibular-related balance performance, cadence during walking with eyes closed, disability level, and overall quality of life. Collectively, these results suggest that a task-oriented, multisensory, and functionally progressive rehabilitation approach may provide clinical benefits compared with conventional home-based vestibular exercise programs.

### 4.1. Dizziness and Vertigo

Dizziness and vertigo are the primary manifestations of vestibular disorders [26], and vestibular rehabilitation remains the cornerstone of conservative treatment. Exercise-based protocols grounded in adaptation, substitution, and habituation principles consistently reduce symptom frequency and severity. For example, Cohen et al. demonstrated that a four-week home-based head movement program significantly decreased vertigo severity and frequency while improving functional independence in patients with chronic peripheral vestibular dysfunction [26]. Similarly, Hillier et al. reported that vestibular rehabilitation is superior to control or no intervention in reducing dizziness frequency in unilateral peripheral vestibular dysfunction [27].

The present findings align with this established evidence while suggesting that TOCT may further optimize symptom resolution. Despite mild baseline vertigo severity and episode frequency of 1–5 per week, vertigo complaints were no longer reported by most patients following TOCT, accompanied by a marked reduction in dizziness. Embedding vestibular stimuli within functional, goal-directed motor tasks may enhance tolerance to symptom-provoking movements encountered in daily life. Rather than relying solely on isolated vestibular exercises, TOCT exposes patients to context-specific challenges that may facilitate more efficient adaptation during real-world activities.

Overall, TOCT achieved outcomes consistent with established vestibular rehabilitation approaches and may represent a structured alternative for symptom management in PVH.

### 4.2. Balance and Sensory Integration

Targeting impaired sensory systems is central to improving balance in vestibular disorders. Organ-specific physiotherapy has been shown to enhance balance and gait by stimulating dysfunctional vestibular structures [28]. Systematic reviews further support the effectiveness of vestibular rehabilitation in improving postural stability and gaze control in PVH [29]. In addition, structured rehabilitation programs have demonstrated significant improvements in balance and disability outcomes across vestibular conditions [30]. Randomized evidence also indicates that activity-based vestibular home programs improve sensory organization test performance and composite balance scores compared with conventional exercise protocols in chronic PVH [31].

In the present study, improvements were particularly pronounced in anterior–posterior (AP) stability parameters. Reduction in AP sway is clinically meaningful in this population, as vestibular hypofunction frequently results in sagittal plane instability due to impaired vestibulo-spinal control. Excessive AP oscillation is associated with increased fall risk and difficulty performing forward–backward weight transfers. Therefore, the observed decrease in AP sway suggests improved postural regulation and safer dynamic balance.

The TOCT protocol was designed to stimulate multiple vestibular end organs simultaneously through functional activities: head movements activated semicircular canals, vertical dynamic tasks challenged saccular pathways, and ambulation-based exercises engaged utricular input. Improvements across most balance parameters support the interpretation that TOCT promoted enhanced multisensory integration rather than isolated sensory adaptation.

From a motor learning perspective, repeated practice of task-specific balance activities may reinforce sensorimotor coordination and support central compensation processes. Although neurophysiological mechanisms were not directly measured, the combined activation of vestibular pathways within meaningful motor contexts may have contributed to improved postural stability, particularly in the sagittal plane.

### 4.3. Gait

Gait disturbances are common in vestibular dysfunction and tend to worsen under conditions that limit visual input or increase head movement demands [32,33,34,35]. Patients typically demonstrate reduced speed, cadence, and stride length as compensatory strategies to maintain stability.

In this study, cadence increased significantly in the TOCT group across walking conditions, whereas gait speed remained unchanged. This pattern may reflect an adaptive strategy characterized by shorter, more frequent steps to enhance dynamic stability. Exposure to functional ambulation tasks under varying sensory demands may have improved temporal gait regulation and step consistency. These findings suggest enhanced dynamic stability under increased sensory demand rather than improvements in locomotor efficiency.

### 4.4. Disability Level and Quality of Life

Vestibular symptoms substantially restrict daily functioning, social participation, and occupational engagement, contributing to increased disability and reduced quality of life. Neuhauser et al. reported that recurrent dizziness and vertigo are associated with significant disability and limitations in activities of daily living [36]. In the present study, both groups demonstrated moderate disability and reduced quality of life at baseline.

Previous reviews confirm that vestibular rehabilitation reduces disability and improves quality of life, balance, and mobility in PVH [1,4,37]. Recent clinical evidence further supports the finding that structured vestibular rehabilitation programs significantly enhance disability scores and health-related quality of life outcomes [30].

Building on this literature, TOCT resulted in clinically meaningful reductions in disability accompanied by significant improvements in overall quality of life. Participants initially presented with functional limitations during rapid head movements, bed mobility, and walking, often leading to activity avoidance. Following TOCT, improvements were observed not only in physical domains but also in emotional well-being. Improvements in symptom and functional performance may have contributed to greater participation in daily and social activities.

Although evidence regarding the use of TOCT in vestibular disorders is limited, findings from neurological populations may provide useful insights into the potential mechanisms underlying the observed improvements. Previous studies in patients with multiple sclerosis, Parkinson’s disease and stroke have shown that TOCT can significantly improve balance, gait performance, and functional mobility by promoting repetitive practice of goal-directed motor tasks and enhancing sensorimotor integration [6,7,8,9]. These improvements have been attributed to principles of motor learning, including task specificity, high repetition, and progressive challenge, which facilitate neuroplastic adaptations within the central nervous system. In the context of peripheral vestibular hypofunction, similar mechanisms may contribute to improved postural control and functional mobility by promoting more efficient integration of vestibular, visual, and somatosensory inputs during real-life activities. Therefore, although the underlying pathophysiology differs between neurological disorders and vestibular dysfunction, the functional training principles embedded within TOCT may support central compensation processes and improve functional performance in patients with PVH [6,7,8,9].

By embedding vestibular rehabilitation within meaningful, real-life tasks, TOCT appears to address both the physical and psychosocial consequences of vestibular dysfunction. These findings support TOCT as a comprehensive intervention for reducing disability and enhancing quality of life in patients with PVH.

### 4.5. Strengths and Limitations

A major strength of this randomized controlled trial is its originality. Although TOCT has been extensively studied in neurological populations, this study is, to our knowledge, the first to examine its application in patients with PVH. The intervention was designed to integrate balance and gait tasks with progressively increasing sensory and motor demands, reflecting real-life movement challenges and aligning with established motor learning and neuroplasticity principles.

Another strength is the multidimensional assessment strategy, which simultaneously captured symptom severity, functional performance, and patient-reported quality of life. This comprehensive approach enabled a more integrated interpretation of treatment effects, extending beyond symptom reduction to functional and participatory outcomes.

Several limitations should be acknowledged. Although clinical symptoms are similar in unilateral and bilateral PVH, the relatively small and heterogeneous sample may limit subgroup-specific inferences. In addition, the short intervention duration and lack of follow-up assessments preclude conclusions regarding the long-term persistence of treatment effects. Future studies should address these limitations by including larger, more homogeneous samples and extended follow-up periods. Also, the statistical analysis was conducted using a per-protocol approach due to missing post-intervention data from participants who withdrew from the study. Future studies with larger samples and complete follow-up data should consider applying intention-to-treat analyses to further strengthen the robustness of the findings. Another limitation of this study is the relatively wide age range of participants and the variability in disease duration, which may have introduced sample heterogeneity. Future studies with larger samples may consider subgroup analyses according to disease severity or duration.

## 5. Conclusions

This randomized controlled trial demonstrated significant group-by-time effects favoring TOCT for dizziness, vertigo, vestibular-related balance performance, cadence during eyes-closed walking, disability level, and overall quality of life in patients with PVH. Within-group analyses further supported meaningful improvements in balance and gait-related functional outcomes following the intervention.

Overall, these findings indicate that incorporating balance and gait tasks within a structured, multisensory, and functionally progressive circuit framework can yield clinically relevant benefits for patients with PVH. Task-oriented circuit training may therefore represent a valuable addition to contemporary vestibular rehabilitation programs.

## Figures and Tables

**Table 1:** Task-oriented circuit training program.

Training Component	Task	Sensory Manipulation/Progression
Gaze Stabilization Training	Head-fixed vertical eye movements	Progressed from seated to standing; visual input gradually reduced (eyes open → eyes closed)
	Head-fixed horizontal eye movements	Progression by increasing head/eye movement speed
	Eye-fixed vertical head movements	Vestibular stimulation increased with larger head movement amplitude
	Eye-fixed horizontal head movements	Performed on firm → foam surface
	Head and eye vertical opposing movements	Reduced visual input and increased movement speed
	Head and eye horizontal opposing movements	Progressed from stable → unstable surface
	Saccadic vertical eye movements	Increased target distance and speed
	Saccadic horizontal eye movements	Visual input manipulation (eyes open/closed depending on tolerance)
	Pursuit eye movements (optokinetic stimulus)	Performed with increasing visual complexity
Balance Training	Rolling on the mat	Progression by reducing base of support
	Vertical rotation	Added head movement to increase vestibular demand
	Spinning on a rotating disc	Progressed with reduced visual input
	Standing on balance board	Surface stability gradually reduced
	Jumping	Increased task speed and dynamic balance demand
	Playing darts	Performed on unstable surface when tolerated
	Reaching	Added trunk rotation and reduced visual input
Gait Training	Walking forward	Progressed by adding head movements
	Walking with vertical head movements	Increased vestibular challenge
	Walking with horizontal head movements	Increased speed and reduced visual cues
	‘8’ shape walking	Added directional changes
	Tandem walking	Reduced base of support
	Walking over an obstacle	Increased obstacle height progressively
	Walking while picking an object from the ground	Added trunk flexion and dual-task demand
	Walking on treadmill	Speed gradually increased
	Climbing and descending stairs	Added head movements when tolerated

Each station was performed for 1 min followed by a 1 min rest period. The total duration of a single training session was approximately 50 min, making it comparable to the duration of the control group intervention. Sensory conditions (visual, vestibular, and somatosensory input) were progressively manipulated during the tasks (e.g., eyes open → eyes closed, firm → foam surface, addition of head movements) depending on the participant’s tolerance and safety. Not all tasks were required to be performed with eyes closed; this condition was applied only when clinically appropriate.

**Table 2:** Characteristics of the participants.

**Characteristics of the** **Participants**		**TOCTG** **(M ± sd)**	**CG** **(M ± sd)**	***p* Value**
Age (year)		44.87 ± 9.32	45.66 ± 15.95	0.880 ^+^
Age (year)		44.87 ± 9.32	45.66 ± 15.95	0.880 ^+^
BMI (kg/m^2^)		28.01 ± 4.41	27.86 ± 5.63	0.936 ^+^
Disease duration (months)		13.88 ± 13.04	17.67 ± 18.91	0.254 ^+^
**Characteristics of the** **Participants**		**TOCTG** **n (%)**	**CG** **n (%)**	***p* Value**
Gender	Female	9 (56.3)	7 (58.3)	0.912 ^x2^
	Male	7 (43.7)	5 (41.7)	
Education level	Primary School	8 (50)	4 (33.3)	0.407 ^x2^
	Secondary School	3 (18.7)	5 (41.7)	
	University	5 (31.3)	3 (25)	
Dominant side	Right	15 (93.8)	12 (100)	1 ^a^
	Left	1 (6.2)	0 (0)	
Affected side	Right Unilateral	4 (25)	6 (50)	0.144
	Left Unilateal	10 (62.5)	3 (25)	
	Bilateral	2 (12.5)	3 (25)	

^+^: independent sample *t* test; ^x2^: chi-square test; ^a^: Fisher exact test; M: mean; sd: standard deviation; TOCTG: Task-oriented circuit training group; CG: Control group; BMI: Body max index; *p* < 0.05.

**Table 3:** Changes in dizziness and vertigo parameters over time and between groups.

Outcome	Group	T1 Mean ± SD	T2 Mean ± SD	Change Mean ± SD	Cohen’s d	*p* (Within the Group)	Main Effect for Group *p* F η^2^p	Main Effect for Time *p* F η^2^p	Time × Group *p* F η^2^p
Dizziness Severity VAS (1–10)	TOCTG	5.75 ± 1.84	2 ± 1.41	3.75 ± 2.40	1.562	<0.001 *	0.076	**<0.001**	0.003 *
	CG	5.50 ± 2.31	4.33 ± 1.55	1.16 ± 1.33	0.872	0.012 *	3.414	**40.404**	**11.154**
							0.116	**0.608**	**0.300**
Dizziness Frequency VAS (1–10)	TOCTG	6.68 ± 3.30	2 ± 1.71	4.68 ± 3.13	1.495	<0.001 *	0.613	**<0.001**	0.057
	CG	5.16 ± 3.18	2.66 ± 1.82	2.50 ± 2.46	1.016	0.005 *	0.262	**42.951**	3.978
							0.010	**0.623**	0.133
Vertigo Severity VAS (1–10)	TOCTG	4.37 ± 3.73	0.56 ± 1.20	−3.81 ± 3.44	1.107	<0.001 *	0.591	**0.003**	0.002 *
	CG	2.91 ± 3.36	3.08 ± 2.96	0.16 ± 2.08	0.07	0.787	0.296	**10.481**	**12.485**
							0.011	**0.287**	**0.324**
Vertigo Frequency VAS (1–10)	TOCTG	2.25 ± 2.04	0.31 ± 0.70	1.93 ± 1.69	1.142	<0.001 *	0.315	**0.001**	0.002 *
	CG	2.08 ± 2.93	2 ± 2.29	0.08 ± 0.99	0.08	0.777	1.050	**13.519**	**11.381**
							0.039	**0.342**	**0.304**

Data are presented as Mean ± Standard deviation (SD). * *p* < 0.05 for within the group using paired-samples *t*-test and for interaction (time × group) using analysis of variance (ANOVA). TOCTG: Task-oriented circuit training group; CG: Control group; VAS: Visual analog scale. The values written in bold in the table represent statistically significant results.

**Table 4:** Changes in balance parameters over time and between groups.

Outcome	Group	T1 Mean ± SD	T2 Mean ± SD	Change Mean ± SD	Cohen’s d	*p* (Within the Group)	Main Effect for Group *p* F η^2^p	Main Effect for Time *p* F η^2^p	Time × Group *p* F η^2^p
SOT Somatosensory (AP)	TOCTG	90.56 ± 11.87	97.12 ± 3.11	−6.56 ± 12.25	0.535	0.040 *	0.112	0.060	0.983
	CG	83.91 ± 24.11	90.33 ± 10.29	−6.41 ±22.41	0.286	0.343	2.712	3.860	0
							0.094	0.129	0
SOT Visual (AP)	TOCTG	72.93 ± 20.57	85.93 ± 7.28	−13 ± 22.06	0.589	0.032 *	0.081	**0.047**	0.199
	CG	85.33 ± 16.89	88.25 ± 8.47	−2.91 ± 16.85	0.172	0.561	3.290	**4.331**	1.738
							0.112	**0.143**	0.063
SOT Vestibular (AP)	TOCTG	38.93 ± 23.84	68.06 ± 7.41	−29.12 ± 25.91	1.123	<0.001 *	0.695 0.157 0.006	**<0.001** **15.818** **0.378**	0.020 * **6.153** **0.191**
	CG	47.58 ± 26.90	54.33 ± 20.42	−6.75 ± 20.06	0.336	0.269			
SOT Somatosensory (ML)	TOCTG	96.93 ± 3.90	98.18 ± 2.53	−1.25 ± 4.10	0.304	0.242	0.178 1.915 0.069	0.094 3.026 0.104	0.209 1.663 0.060
	CG	89.75 ± 20.91	98.16 ± 1.80	−8.41 ± 21.85	0.384	0.209			
SOT Visual (ML)	TOCTG	58.93 ± 24.18	71.37 ± 12.15	−12.43 ± 21.59	0.575	0.036 *	0.683 0.227 0.009	**0.004** **9.926** **0.276**	0.847 0.038 0.001
	CG	61.91 ± 32.13	76 ± 24.66	−14.08 ± 22.63	0.622	0.054			
SOT Vestibular (ML)	TOCTG	20.50 ± 22.58	39.31 ± 22.94	−18.81 ± 25.49	0.737	0.010 *	0.587 0.303 0.012	**<0.001** **16.333** **0.386**	0.966 0.002 0
	CG	24.91 ± 24.99	43.33 ± 23.58	−18.41 ± 22.12	0.832	0.015 *			

Data are presented as Mean ± Standard deviation (SD). * *p* < 0.05 for within the group using paired-samples *t*-test and for interaction (time × group) using analysis of variance (ANOVA). TOCTG: Task-oriented circuit training group; CG: Control group; AP: Antero-posterior; ML: Medio-lateral; SOT: Sensory organization test. The values written in bold in the table represent statistically significant results.

**Table 5:** Changes in gait parameters over time and between groups.

Outcome	Group	T1 Mean ± SD	T2 Mean ± SD	Change Mean ± SD	Cohen’s d	*p* (Within the Group)	Main Effect for Group *p* F η^2^p	Main Effect for Time *p* F η^2^p	Time × Group *p* F η^2^p
walking with open eyes (cadence)	TOCTG	113.72 ± 11.64	119.96 ± 10.02	−5.97 ± 9.05	0.659	0.019 *	**0.007**	**0.025**	0.085
	CG	106.15 ± 8.33	106.99 ± 7.61	−0.84 ± 4.64	0.181	0.544	**8.702**	**5.638**	3.200
							**0.251**	**0.178**	0.110
walking with open eyes (speed)	TOCTG	1.32 ± 0.51	1.25 ± 0.17	0.06 ± 0.56	0.107	0.632	0.669	0.970	0.395
	CG	1.18 ± 0.57	1.25 ± 0.56	−0.07 ± 0.13	0.538	0.072	0.187	0.001	0.750
							0.007	0	0.028
walking with closed eyes (cadence)	TOCTG	102.02 ± 10.33	109.84 ± 11.44	−7.82 ± 9.48	0.824	0.005 *	**0.011** **7.463** **0.223**	**0.034** **5.038** **0.162**	0.039 * **4.714** **0.153**
	CG	96.06 ± 10.03	96.19 ± 9.73	−0.13 ± 8.98	0.01	0.961			
walking with closed eyes (speed)	TOCTG	1.68 ± 3.37	0.91 ± 0.20	0.76 ± 3.47	0.219	0.391	0.224 1.555 0.056	0.478 0.519 0.020	0.431 0.639 0.024
	CG	0.68 ± 0.18	0.72 ± 0.24	−0.04 ± 0.22	0.181	0.554			
walking with horizontal head movements (cadence)	TOCTG	105.39 ± 9.61	115.36 ± 9.06	−9.97 ± 12.96	0.769	0.008 *	**0.002** **11.569** **0.308**	**0.003** **10.915** **0.296**	0.212 1.636 0.059
	CG	97.83 ± 12.46	102.24 ± 7.72	−4.40 ± 8.82	0.498	0.112			
walking with horizontal head movements (speed)	TOCTG	1.05 ± 0.16	1.16 ± 0.19	−0.11 ± 0.24	0.458	0.093	**0.021** **6.011** **0.188**	**0.029** **5.329** **0.170**	0.688 0.165 0.006
	CG	0.89 ± 0.29	0.97 ± 0.22	−0.07 ± 0.15	0.466	0.116			
walking with vertical head movements (cadence)	TOCTG	106.53 ± 12.29	119.43 ± 8.41	−12.89 ± 13.86	0.930	0.002 *	**0.004** **10.097** **0.280**	**<0.001** **18.537** **0.416**	0.115 2.657 0.093
	CG	98.95 ± 11.85	104.75 ± 10.39	−5.81 ± 6.63	0.876	0.011 *			
walking with vertical head movements (speed)	TOCTG	2.83 ± 7.03	1.20 ± 0.16	1.63 ± 7.17	0.227	0.376	0.290 1.167 0.043	0.468 0.543 0.020	0.412 0.697 0.026
	CG	0.88 ± 0.30	0.98 ± 0.25	−0.10 ± 0.13	0.769	0.023 *			

Data are presented as Mean ± Standard deviation (SD). * *p* < 0.05 for within the group using paired-samples *t*-test and for interaction (time × group) using analysis of variance (ANOVA). TOCTG: Task-oriented circuit training group; CG: Control group; cadence: step/min; speed: m/s. The values written in bold in the table represent statistically significant results.

**Table 6:** Comparison of disability level and quality of life parameters between the groups.

Outcome	Group	T1 Mean ± SD	T2 Mean ± SD	Change Mean ± SD	Cohen’s d	*p* (Within the Group)	Main Effect for Group *p* F η^2^p	Main Effect for Time *p* F η^2^p	Time × Group *p* F η^2^p
DHI Physical	TOCTG	16.62 ± 5.92	6.43 ± 4.88	10.18 ± 6.89	1.477	<0.001 *	0.226	**<0.001**	0.008 *
	CG	15 ± 5.81	12.83 ± 8.37	2.16 ± 7.97	0.271	0.367	1.536	**19.270**	**8.122**
							0.056	**0.426**	**0.238**
DHI Emotional	TOCTG	15.37 ± 8.02	5.56 ± 3.70	9.81 ± 10.33	0.949	0.002 *	**0.023** **5.864** **0.184**	**0.003** **10.770** **0.293**	0.062 3.799 0.127
	CG	16.50 ± 9.34	14 ± 6.82	2.50 ± 9.38	0.266	0.376			
DHI Functional	TOCTG	23.12 ± 7.76	9.50 ± 8.01	13.62 ± 9.77	1.394	<0.001 *	0.281 1.211 0.045	**<0.001** **20.858** **0.445**	0.012 * **7.291** **0.219**
	CG	20.83 ± 8.46	17.33 ± 8.83	3.50 ± 9.87	0.354	0.245			
DHI Total	TOCTG	55.12 ± 18.54	21.62 ± 15.07	33.50 ± 23.64	1.417	<0.001 *	0.086 3.192 0.109	**<0.001** **19.493** **0.428**	0.013 * **7.187** **0.217**
	CG	52.33 ± 21.68	44.16 ± 21.54	8.16 ± 26.16	0.311	0.303			

Data are presented as Mean ± Standard deviation (SD). * *p* < 0.05 for within the group using paired-samples *t*-test and for interaction (time × group) using analysis of variance (ANOVA). TOCTG: Task-oriented circuit training group; CG: Control group; DHI: Dizziness handicap inventory. The values written in bold in the table represent statistically significant results.

## Data Availability

The data that support the findings of this study are available from the corresponding author, Y.A., upon reasonable request. The data are not publicly available due to privacy and ethical restrictions.

## Abbreviations

The following abbreviations are used in this manuscript:

PVH	Peripheral vestibular hypofunction
TOCT	Task-oriented circuit training
TOCTG	Task-oriented circuit training group
CG	Control group
RPE	Rating of Perceived Exertion
VAS	Visual Analog Scale
SOT	Sensory Organization Test
AP	Anterior–Posterior
ML	Medial–Lateral
DHI	Dizziness Handicap Inventory
